# Enhancing Documentation of Upper Limb Specialist Team Meetings at a Tertiary Trauma Centre

**DOI:** 10.7759/cureus.97169

**Published:** 2025-11-18

**Authors:** Uday Mahajan, Moheeb Gadullah, Raghunaathan Rangan, Kashif Memon

**Affiliations:** 1 Trauma and Orthopaedics, Queen Elizabeth Hospital Birmingham, Birmingham, GBR

**Keywords:** clinical governance, electronic patient record, quality improvement initiative, specialist team meeting, trauma and orthopaedics

## Abstract

Background: Specialist team meetings play a key role in the management of complex trauma and reconstructive cases, but their impact depends on accurate documentation. Inconsistent or informal record-keeping risks duplication, transcription errors, and loss of critical information, with implications for governance and patient safety.

Methods: A quality improvement project was undertaken in the upper limb specialist team meeting at a tertiary trauma centre. At baseline, outcomes were recorded as PowerPoint slides stored in a shared folder without systematic entry into the electronic patient record (EPR). Interventions included the introduction of a structured documentation template, centralised storage, and a shared worklist integrated into the clinical portal. A re-audit of all meetings between 16 May and 22 November 2024 assessed completeness of documentation, reasons for omissions, and qualitative benefits for patient care.

Results: At baseline, no complex patient outcomes were documented in the EPR. Following the intervention, 44 complex cases were reviewed, of which 39 (89%) had outcomes recorded directly in the EPR through the shared worklist. The five undocumented cases occurred during the junior doctor changeover period, highlighting the importance of robust handover processes. The new system also improved accessibility, facilitated follow-up, and strengthened clinical governance by creating a permanent, retrievable record of decisions.

Conclusion: Structured, EPR-integrated documentation substantially improved the completeness and accessibility of specialist team meeting records, supporting safer patient handover and stronger governance. These findings align with international evidence that high-quality electronic records improve care quality and suggest that similar frameworks could be applied across subspecialty services to standardise documentation and enhance patient safety.

## Introduction

Specialist team meetings are an established component of modern hospital practice, providing a structured forum for consultants to review complex cases, share expertise, and agree on treatment plans [1,2]. Their value depends not only on the quality of the discussion but also on how outcomes are documented and communicated [3]. Accurate records ensure that agreed management plans are accessible to all members of the clinical team, support continuity of care, and provide an auditable record for governance and medico-legal purposes [4]. However, suboptimal documentation in trauma and orthopaedics, including ward rounds and trauma meetings, has been identified as a critical issue leading to clinical incidents and compromising patient safety [5,6].

Despite their importance, documentation practices in such meetings are often inconsistent. Outcomes may be recorded informally, stored outside the electronic patient record (EPR), or left to individual clinicians to transcribe retrospectively. This creates risks of duplication, transcription error, and loss of critical information. Poorly structured records also reduce visibility for wider clinical teams, limit accountability, and complicate patient handover [7]. Indeed, research consistently highlights that substandard documentation within perioperative settings, as well as during trauma meetings, directly compromises patient safety and the seamless transfer of information between multidisciplinary healthcare teams [8-10].

At our tertiary trauma centre, the upper limb specialist team meeting was previously documented using PowerPoint slides (Microsoft Corp., Redmond, WA) stored in shared folders, without systematic linkage to the EPR. This created barriers to accessibility and continuity, undermining the ability to track complex patients over time. To address these shortcomings, we introduced a structured documentation process integrated into the clinical portal, aiming to improve accuracy, accessibility, and governance.

This paper reports the findings of two audit cycles evaluating the effect of this intervention on the completeness of documentation and its impact on patient care processes.

## Materials and methods

This project was undertaken as a quality improvement initiative within the upper limb specialist team meeting at Queen Elizabeth Hospital Birmingham, a tertiary trauma centre in Birmingham, England. The meeting is held weekly, either in person or virtually, and is attended by upper limb consultants and a clinical fellow. Cases typically include complex trauma, metalwork-related problems, and other reconstructive challenges, with the outcomes of the meeting guiding subsequent patient management.

A baseline assessment in May 2025 reviewed existing documentation practices. At that time, outcomes were recorded as PowerPoint files stored in a shared folder, with no systematic entry into the EPR (Prescribing, Information and Communication System (PICS)/clinical portal). This process carried a risk of transcription errors and limited accessibility for wider clinical teams, and provided no clear mechanism for tracking complex patients. No standardised template was in use, and the responsibility for documentation varied between individuals. The structured template included fields for patient identifiers, diagnosis, key discussion points, agreed management plan, responsible consultant, and follow-up actions.

To address these shortcomings, a set of interventions was introduced. These included the development of a structured template for recording outcomes, the creation of a centralised folder for storing minutes, and communication with consultants to raise awareness of the new process. A shared worklist was also established within the clinical portal, enabling the collation of complex cases in a single location. 

Following these changes, a re-audit was conducted covering all specialist team meetings held between 16 May and 22 November 2024. During this period, documentation of outcomes was moved directly into the EPR, supported by the dedicated worklist, to ensure that decisions were linked to individual patient records and available for ongoing care. All cases discussed in this timeframe were reviewed. The primary outcome measure was the proportion of cases with outcomes documented in the EPR. Secondary outcomes included the identification of missing cases, exploration of reasons for omission, and qualitative assessment of the shared worklist’s impact on patient monitoring, handover, and governance.

Data were analysed descriptively using Microsoft Excel (Microsoft Corp.). Categorical variables, such as documentation completeness, were expressed as frequencies and percentages. Given the binary nature of the data and small sample size, Fisher’s exact test was used to compare documentation rates before and after the intervention. A p-value of <0.05 was considered statistically significant. This project was registered as a quality improvement audit within the institution and did not require formal ethics committee approval. No identifiable patient data were collected or reported.

## Results

At the baseline review in May 2025, none of the complex cases discussed at the upper limb specialist team meetings were systematically documented in the EPR. Outcomes were instead recorded as PowerPoint slides stored in a shared folder. This approach carried risks of transcription errors, poor accessibility for the wider clinical team, and a lack of a central mechanism to track complex patients. Responsibility for documentation varied between individuals, and no structured template was in use, resulting in inconsistent and incomplete records.

Following the introduction of standardised protocols, centralised storage, and the creation of a shared worklist within the clinical portal, documentation practices improved substantially. In the re-audit period between 16 May and 22 November 2024, a total of 44 complex cases were discussed. Of these, 39 cases (89%) had outcomes documented directly in the EPR through the dedicated worklist. The five undocumented cases all occurred in August 2024, coinciding with the junior doctor changeover period, suggesting that staff transition contributed to lapses in documentation. A Fisher’s exact test comparing documentation completeness before and after the intervention (0% vs 89%) demonstrated a statistically significant improvement (p < 0.001).

The move to direct EPR entry also brought qualitative benefits. The shared worklist enabled consultants to monitor cases in real time, facilitated follow-up of complex patients, and improved handover by collating outcomes in a single, accessible location. Incorporating decisions into the patient record also strengthened clinical governance by ensuring that outcomes were auditable, retrievable, and consistent across the team. The changes are summarised below in Table 1.

**Table 1:** Comparison of documentation outcomes before and after process change EPR: electronic patient record

Parameter	Baseline (May 2025)	After Intervention (May–Nov 2024)	Change/Comment
Total complex cases discussed	Not systematically recorded	44	–
Cases documented in EPR	0 (0%)	39 (89%)	Significant improvement
Cases not documented	All	5	All five occurred during the junior doctor changeover
Method of record-keeping	PowerPoint slides in shared folder	Direct EPR entry via shared worklist	Introduced standardised protocol
Accessibility of records	Limited	Readily available in EPR for all consultants	Improved visibility and handover
Governance alignment	Informal, no audit trail	Fully auditable within EPR	Strengthened clinical governance

## Discussion

This project demonstrated that introducing a structured, EPR-integrated process for documenting upper limb specialist team meetings at a tertiary trauma centre markedly improved record-keeping. At baseline, outcomes were stored as PowerPoint slides in shared folders with no direct entry into the EPR. This informal approach was prone to transcription errors, lacked accessibility, and created uncertainty about responsibility for documentation. Following the intervention, 89% of cases were recorded directly in the EPR, providing a clear and auditable record of decisions. The few omissions observed coincided with the junior doctor changeover period, emphasising the need for robust handover arrangements to maintain compliance.

Our findings mirror those of wider studies examining record-keeping in multidisciplinary meetings. Khumalo and Kane reported that although electronic documentation is becoming standard, there remain gaps in standardisation, responsibility allocation, and verification of records [11]. Similarly, Kane and Luz described the challenge of capturing not only the agreed outcome but also the reasoning and knowledge generated during case discussions [12]. While the new documentation system improved completeness and accessibility, it currently records only final decisions rather than the underlying rationale or differing opinions. Capturing this contextual information in future iterations would enhance transparency, align with recommendations by Kane and Luz, and increase the educational value of meeting records.

Completeness and accessibility are critical. Zegers et al. showed that poor quality or incomplete records are associated with higher adverse event rates, making record-keeping a proxy for quality of care [13]. Our project demonstrated that structured EPR entry reduced the risk of omissions and created a permanent, retrievable record, thereby strengthening patient safety and governance. However, as highlighted in the literature, capturing the rationale and alternative options discussed would further enhance transparency and educational value [11,12].

Scaling documentation improvements beyond a single team raises additional challenges. Christiansen et al. identified responsibility allocation, documentation routines, and access control as barriers when implementing shared EHR systems across institutions [14]. Our intervention, confined to one service within one hospital, avoided issues of dual documentation and access integration. Nevertheless, wider adoption would need to consider these governance and legal implications, particularly if a standardised framework were implemented across specialities or trusts.

In addition to improving governance and safety, the structured documentation process produced tangible operational benefits. The dedicated worklist in the clinical portal enabled consultants to track complex patients, plan follow-up, and hand over cases more reliably, demonstrating how structured electronic records can translate broader principles of coordinated care into daily clinical practice [13].

This study has limitations. It was conducted in a single specialist team over a short period, with relatively small case numbers. The outcomes measured focused on documentation completeness rather than direct patient outcomes, though the link between record quality and care quality is supported in the literature [14]. Despite these limitations, the improvement from 0% to 89% documentation demonstrates the value of structured processes, and the approach could be adapted by other services. Brief lapses during staff changeover highlight the need for structured induction, clear responsibility, and checklist-based handover to maintain documentation continuity.

## Conclusions

Introducing a structured, EPR-integrated process for documenting upper limb specialist team meetings transformed record-keeping at our tertiary trauma centre. Documentation improved from no systematic EPR entries at baseline to 89% of cases captured after the intervention, with additional benefits in patient monitoring, follow-up, and governance.

These findings are consistent with international evidence that structured electronic documentation enhances safety, continuity, and accountability, while informal or inconsistent records increase the risk of error. Challenges remain, particularly around ensuring continuity during staff transitions and capturing not only outcomes but also the rationale for decisions.

Future work should focus on embedding structured documentation frameworks across subspeciality services, exploring options for including decision rationale and patient factors, and addressing wider governance and integration challenges as highlighted in shared electronic health record systems. By doing so, the benefits demonstrated in this project can be scaled to support safer and more transparent care across the health system.
